# Continuous quality improvement interventions to improve long-term outcomes of antiretroviral therapy in women who initiated therapy during pregnancy or breastfeeding in the Democratic Republic of Congo: design of an open-label, parallel, group randomized trial

**DOI:** 10.1186/s12913-017-2253-9

**Published:** 2017-04-26

**Authors:** Marcel Yotebieng, Frieda Behets, Bienvenu Kawende, Noro Lantoniaina Rosa Ravelomanana, Martine Tabala, Emile W. Okitolonda

**Affiliations:** 1 Division of Epidemiology, The Ohio State University, College of Public Health, 304 Cunz Hall, 1841 Neil Avenue, Columbus, OH 43210 USA; 2 Department of Epidemiology, The University of North Carolina at Chapel Hill, Chapel Hill, NC USA; 3 Department of Social Medicine, The University of North Carolina at Chapel Hill, Chapel Hill, NC USA; 4 The University of Kinshasa, School of Public Health, Kinshasa, Democratic Republic of Congo

**Keywords:** Universal test-and-treat, Prevention of mother-to-child transmission, Continuous quality improvement, Cluster-randomized trial, DR Congo

## Abstract

**Background:**

Despite the rapid adoption of the World Health Organization’s 2013 guidelines, children continue to be infected with HIV perinatally because of sub-optimal adherence to the continuum of HIV care in maternal and child health (MCH) clinics. To achieve the UNAIDS goal of eliminating mother-to-child HIV transmission, multiple, adaptive interventions need to be implemented to improve adherence to the HIV continuum.

**Methods:**

The aim of this open label, parallel, group randomized trial is to evaluate the effectiveness of Continuous Quality Improvement (CQI) interventions implemented at facility and health district levels to improve retention in care and virological suppression through 24 months postpartum among pregnant and breastfeeding women receiving ART in MCH clinics in Kinshasa, Democratic Republic of Congo. Prior to randomization, the current monitoring and evaluation system will be strengthened to enable collection of high quality individual patient-level data necessary for timely indicators production and program outcomes monitoring to inform CQI interventions. Following randomization, in health districts randomized to CQI, quality improvement (QI) teams will be established at the district level and at MCH clinics level. For 18 months, QI teams will be brought together quarterly to identify key bottlenecks in the care delivery system using data from the monitoring system, develop an action plan to address those bottlenecks, and implement the action plan at the level of their district or clinics.

**Discussion:**

If proven to be effective, CQI as designed here, could be scaled up rapidly in resource-scarce settings to accelerate progress towards the goal of an AIDS free generation.

**Trial registration:**

The protocol was retrospectively registered on February 7, 2017. ClinicalTrials.gov Identifier: NCT03048669.

## Background

In 2013, the World Health Organization (WHO) recommended lifelong triple antiretroviral therapy (ART) to all HIV-positive pregnant and breastfeeding women regardless of CD4 count (Option B+) [1]. By 2015, 91% of the 1.1 million women receiving antiretroviral medicines for prevention of mother-to-child transmission of HIV (PMTCT) were on lifelong ART. [2] However, because of high rate of loss-to-follow up (LTFU) and poor quality of care across the continuum of HIV care in Maternal and Child Health (MCH) clinics [3–5], over 150,000 children were newly infected [6]. Without substantial improvement in the quality of HIV care in MCH clinics, particularly in the resource-poor settings, the global goal of an AIDS free generation might not be achieved even if all pregnant women were tested and initiated on ART. [7]

Numerous interventions have been proposed to address the low performance of HIV care delivery system in MCH clinics. However, virtually all of those interventions focus on a single component of the healthcare system performance: the patient [8]. Organizational factors such as availability of an effective supply chain management, regular supervision and support, schedule of services, use of standard operational procedures, staff-patient ratio, model of care; provider factors (e.g. training, satisfaction and support) and patient-level factors (e.g., sociodemographic, perceived and measured health, HIV knowledge, distance to the clinics, and availability of psychosocial support) and their interrelationship are key determinants of healthcare delivery system performance [8]. A package of interventions aimed at each component of the service triad (healthcare delivery systems, providers, and beneficiaries), that can be easily scaled-up in resource-poor settings, is needed to produce the rapid gain in service delivery performance that is required for the elimination of the mother-to-child transmission of HIV.

Quality Improvement (QI) Collaborative is one of the most popular methods for organizing sustained improvement efforts at hospitals and ambulatory practices worldwide. In the Breakthrough Series approach, this is also referred to as continuous quality improvement (CQI) [9], where QI teams from multiple sites across a region or country are brought together to focus on a common problem. However, despite its popularity, CQI effectiveness has never been demonstrated in a randomized trial or a well-designed comparative study [10]. In a large non-randomized comparative study evaluating the effectiveness of CQI interventions in improving the quality of care for HIV-infected patients, in clinics receiving funding from the Ryan White Comprehensive AIDS Resources Emergency Act in the United States of America [11], the intervention did not significantly increase the proportion of patients with a suppressed viral load. However, since 2011, South Africa has experienced a remarkable improvement across all key indicators in the PMTCT cascade [12], attributed, at least in part, to the QI initiatives implemented at facility level [12–16]. But whether the results can be reproduced in poorer settings in sub-Saharan Africa, where the existing health infrastructure including capacity for strong monitoring and evaluation are often weak, remain to be shown.

The aim of our study is to evaluate the effectiveness of CQI interventions, implemented at facility level using participatory data-driven approaches, on-site monitoring, and supervisory support, on long term retention in care and sustained virological suppression among pregnant and breastfeeding women who start lifelong ART in MCH clinics. A pragmatic, open label, parallel, group randomized design will be used. In this paper, we want to describe the methods we plan to use to this effect.

## Methods

### Study setting

The proposed study will be implemented in Kinshasa, the capital city province of the Democratic Republic of Congo (DRC). DRC is one of the 21 sub-Saharan Africa’s with the largest unmet need for PMTCT [7]. DRC’s National AIDS Strategic Plan goal calls for the virtual elimination of MTCT (MTCT rate <5%) by 2017. Starting in October 2013 and with support from the United States President's Emergency Plan for AIDS Relief (PEPFAR) and the Global Fund to Fight AIDS, Tuberculosis and Malaria, DRC has been progressively scaling-up Option B+ (ART for life to all HIV+ pregnant women). Roughly 9 in 10 pregnant women in DRC attend at least one antenatal care (ANC) visit before delivery, surpassing 97% in Kinshasa [17, 18]. For over a decade, through a strong collaboration between the Kinshasa School of Public Health (KSPH), the Ministry of Health, the main faith-based organizations that run most of the primary healthcare clinics in Kinshasa [i.e. the Salvation army (Armée du Salut) and the Catholic Health Board (Bureau Diocésain des Oeuvres Médicales (BDOM)], our team has provided technical support for MCH clinics in Kinshasa and elsewhere in DRC for the implementation of HIV prevention, care and treatment programs including PMTCT [18–32]. In 2010, as part of this technical support, our team developed and supported the implementation of a mother-infant register to help monitor PMTCT care provided in MCH clinics via collection of key program indicators. The registry was modeled after the WHO’s *Three Interlinked Patient Monitoring Systems for HIV Care/ART, MCH/PMTCT (including malaria prevention during pregnancy), and TB/HIV: Standardized Minimum Data Set and Illustrative Tools* and has been adapted by the National AIDS Program for the monitoring of Option B+ across the country.

### Randomization

Although the interventions will be implemented mainly at the clinic level, because the first level of supervision for MCH clinics is the Health District Bureau, and to avoid potential contamination of the control clinics, the unit of randomization with be the health district. All 35 health districts in Kinshasa will be eligible. Health districts will be stratified by urban and peri-urban location and randomized to CQI or standard of care using a computer random generated number and the list of district in each stratum ordered alphabetically.

### Eligibility criteria

In each district, the top three MCH clinics in terms of number of HIV-infected pregnant women served in 2015 and 2016 will be selected. All HIV-infected pregnant and breastfeeding women receiving care at any participating clinics during the study period and their HIV-exposed infants will be eligible for enrollment. Participants will be excluded if they refuse to participate in the study. A written consent will be obtained from each participating woman as well as a written permission for the participation of their HIV exposed infant (HEI).

### Study implementation

The study will be implemented in two phases. In Phase I, to inform the design of CQI interventions, we will collect information on the key characteristics of the healthcare delivery system in the facilities eligible for the study. We will also strengthen the current monitoring and evaluation system in MCH clinics to insure that both programmatic and clinical outcome indicators that will be needed for the CQI implementations will be timely available before randomization occurred.

### Interventions

The package consists of CQI interventions implemented at facility level using participatory data-driven approaches, on-site monitoring, and supervisory support. In health districts randomized to intervention, we will set up a quality improvement team at the level of the district that includes the supervisor from the health district Bureau and the head of PMTCT from each of the participating MCH clinics, and at the level of each clinic. A clinic level QI team will include at least one staff each from ANC, delivery/maternity, and well-child services. A study staff member will serve as rapporteur of each QI team. Immediately following randomization, we will bring together QI teams in a half a day meeting during which they will review monitoring data on program and clinical outcome indicators and identify key bottlenecks in the care delivery system that is affecting those outcomes. The group will then develop and agree on an action plan to modify the priority bottlenecks as well as the key indicators to include in the quarterly data for actions reports that will be used to monitor the changes resulting from the implementation of the action plan. QI teams will be responsible for the implementation of the action plan at the level of their respective clinics. A member of the health district QI team and a study team member will visit each participating clinic at least once a month to supervise and support the implementation of the action plan. Every 3 months, using data from the monitoring system, the study team will generate a quarterly report including a color-coded dashboard for tracking changes using the ‘traffic light’ approach. An indicator will score green when the target has been achieved, yellow/amber when there is a progress from the baseline value without reaching the target, and red when there is no progress or downward movement. The report will be generated for each participating clinic in the intervention group and shared with the QI teams and the management of the clinic. QI teams will be brought together to review these indicators, share experience, identify remaining gaps in the service delivery, and develop a new priority action plan for the next quarter in cycle for 18 months. To limit possible contamination, all staff from a randomized district/clinic who may have a dual appointment in another facility will be excluded from QI teams.

### Standard of care

In health districts randomized to standard of care, the same strengthening of the data collection system for the monitoring of indicators as in the intervention group will be implemented. At least once a month, a study staff will visit each clinic irrespective of their randomization to extract information for the mother-infant register into an electronic database. No report on indicators will be produced for these clinics for the duration of the study. Staff from clinics and health district bureau in the standard of care group will not be associated with the quarterly review of the indicators. The study will not interfere with any other HIV service provision activity in the standard of care group.

### Masking

By the nature of the intervention, it will not be possible to mask the intervention to clinics and study staffs. However, participants (HIV-infected mothers) will not be informed about the randomization nor the intervention.

### Outcomes

Two primary outcomes will be considered: loss-to-follow-up (the proportion of participants for whom the whereabouts is unknown at the evaluation time) and virological suppression (proportion of participants with undetectable viral load). Several secondary outcomes will also be considered including timely infant HIV diagnosis, retention in care, non-adherence to scheduled visits, virological suppression, MTCT rates, and survival (see Table 1 for definitions). Indicators used for the dashboard will also be analyzed as secondary outcomes.

**Table 1:** Outcomes and definitions

Outcomes	Definition
Primary	
Loss-to-follow-up	Proportion of participants for whom the whereabouts is unknown at: delivery, 6 weeks, 12 and 24 months postpartum
Virological suppression	Proportion of participants with undetectable viral loat at: delivery, 12 and 24 months postpartum.
Secondary	
Timely infant HIV diagnosis	Proportion of HIV-exposed infant with an appropriate HIV test result at 6 weeks, 12 and 24 months postpartum.
Timely ART initiation	Proportion of HIV-infected participants (mother and infant) initiated on ART within 2 weeks of diagnosis
Retention in care	Proportion of participants who are known to be receiving HIV care 6 weeks, 12 and 24 months postpartum.
Non-adherence to scheduled visits	Proportion of participants whose whereabouts is unknown or who are in care but have missed at least one scheduled visit over the evaluation period at: delivery, 6 weeks, 12 and 24 months postpartum.
Virological suppression	Proportion of participants with undetectable viral load at: delivery, 12 and 24 months postpartum.
MTCT rates	Proportion of infants born to participating women who tested positive for HIV at 6 weeks, 12 and 24 months postpartum.
Survival	Proportion of participants known to be alive at 6 weeks, 12 and 24 months postpartum.

### Sample size justification

Our sample size estimation is based on primary outcome LTFU. The minimum sample size to detect the expected difference of proportions assuming individual randomization with a level of significance set at 95% (2-sided) and at 80% power was first calculated and adjustment was made by inflating the individually randomized sample size by a factor given by $d=1+\rho \left(\overline{m}-1\right),$ where $\overline{m}$ is the average cluster size (10) and ρ the internal variability that represents how strongly individuals within clusters are related to each other (intracluster correlation (ICC)) [33]. Inflating the individually randomized sample size by this factor counteracts the loss of power due to a clustered design [34]. Analysis of data from a previous trial in some of those clinics showed ICC for various outcomes ranging from 0.20 to 0.30 [35]. Using data from our trial on conditional cash transfer which showed a 20.3% LTFU at 6 weeks postpartum in the control group [36], assuming that by 24 months postpartum, at least 30% of participants would have experienced the outcome in the control group and that the CQI interventions will be considered effective if there is an absolute reduction in LTFU by 30% at 24 months (namely a proportion of LTFU of 21%) in the intervention group, we determined that 367 mother-infant pairs will be needed in each arm under individual randomization. Adjusting for the inflation factor, under a conservative ICC of 0.30, we will need to enroll 1358 participants per arm or 2716 HIV-infected women.

### Data collection

Data for primary and secondary outcome will be collected via the mother-infant register. The following key information is documented in the register: a maternity code that is used to link mother and infant, address and telephone number, gestational age, date of birth, date of reference to HIV care and treatment if any and location of the referral, and other information necessary to calculate PEPFAR’s PMTCT indicators including information on infant feeding practices, cotrimoxazole prophylaxis at subsequent visits, date of first sample collection for early infant diagnosis at 6 weeks and the result, a second sample collection and result at 9 months, and eventually a serological test at 18 months for all infants with HIV-negative PCR results is also documented. At least once a month, a study nurse equipped with a laptop will visit each participating clinic to extract information from the register into an electronic database.

Data on healthcare system delivery characteristics will be collected via a survey conducted at the beginning of the study and at the end of the study (to assess change). The Facility Audit of Service Quality (FASQ) will be adapted to assess the key organizational characteristics of HIV care (including lifelong ART) delivery in participating MCH clinics. FASQ is a relatively low cost approach developed, by MEASURE Evaluation, for district level monitoring or service availability and quality [37]. It provides information on the type of services; status and functionality of infrastructure, equipment and quality of care. The key areas covered include: 1) the range of services offered (HIV testing and counseling, lifelong ART, laboratory), staffing and staff qualifications, operating hours, community linkages, selected administrative and quality control procedures; 2) Facility infrastructure (privacy/capacity, laboratory, delivery, well-child clinics); 3) Readiness to provide quality care in six areas: family planning; STI management; antenatal care; maternal/delivery care and post abortion care; child health/welfare; and HIV prevention, treatment, and care; and the digital maps of facilities and services available.

Data on individual-level patient characteristics including: socio-demographic and economic characteristics, beliefs about the health services (cost, quality), cognitive factors (knowledge about HIV and ART, perceived susceptibility and perceived benefit of early ART, perceived self-efficacy for adherence, perceived need for ART and other HIV services), psychological factors (e.g. depression, fear of stigma), social factors (social support – friends, family, male partner involvement in MCH care, intimate partner violence), and accessibility to the clinics (distance, transportation, waiting time) will also be collected through face to face interviews at enrollment (within 1 month of ANC registration), after delivery (2–3 days in postpartum ward), at 6 weeks (6 weeks post-partum visit), at 24 weeks (end of exclusive breastfeeding period), and every 6 months thereafter through the end of all breastfeeding exposure at 24 months. Each completed questionnaire will be verified by a study supervisor for completeness and consistency immediately after the interview. Questionnaires with missing or inconstant responses will be returned to the interviewer for correction before data entry. The distribution of each variable in the database will be generated on regular basis to also check for completeness and consistency.

### Statistical analyses

The frequencies of outcomes will be compared between the intervention and control groups using adjusted Pearson’s Chi-square [33]. All analyses will be intent-to-treat, meaning that women will be kept in their randomized group independently of whether they actually received all their care in the group. The intervention and control groups will be compared for baseline prognostic factors and any imbalances that persist after randomization will be adjusted for using logistic regression. Within district- and clinic-level clustering will be accounted for using the generalized estimating equation.

### Ethical consideration

the study is approved by The Ohio State University institutional review board (Study ID: 2015H0440) and the Kinshasa School of Public Health Ethical committee. All participants will provide their signed informed consent. Parental permission will be obtained before enrollment of HIV-exposed infants. The protocol was retrospectively registered to ClinicalTrials.gov on February 7, 2017, 2 months after the beginning of enrollment but before randomization. : NCT03048669.

## Discussion

The proposed study has multiple strengths. 1) The comprehensive approach of the intervention in its ability to affect all three pillars of a health system delivery performance: healthcare delivery systems, providers, and beneficiaries (patients). Although there are a multitude of trials evaluating different strategies to improve retention in care and untimely the long-term outcomes of HIV care in MCH clinics, virtually all of them focused on the single patient components. 2) The rigorous pragmatic design. Contrary to the only other randomized trial currently evaluating CQI in Nigeria [38], for which clinics were only eligible if they met a minimum set of organizational criteria such as capacity to provide ART until 12–18 months postpartum, provision of postpartum care follow-up for HIV+ women, or availability of, at least, 2 trained community health extension workers to be eligible, the only requirement to be part of this study is to be one of the top three PMTCT providers in the health districts. 3) The Potential for high impact: if the CQI interventions are proven to be effective in improving long-term outcomes of lifelong ART in MCH clinics, it will provide the DRC government and PEPFAR with a scalable strategy to strengthen the quality of HIV continuum of care and help the country and PEPFAR move towards their aspiration of an AIDS-free generation.

A source of concern for the proposed study is the potential for crossover. This is an open label implementation science study that is being conducted in collaboration of the Ministry of Health through the National and Provincial AIDS Control Program, the main organizations that run most of the health care facilities in Kinshasa (BDOM, Armée du Salut), as well as their PMTCT implementing partners. Although we have their support for the study, if through the study they have the impression that one intervention developed within the CQI could be of particular help in clinics randomized to the control condition, it is possible they may intentionally or inadvertently carry that intervention in other clinics they are responsible for. If this were to happen, we have planned for robust data collection in the pre-randomization phase on the outcomes to allow for a pre- and post-intervention comparison of outcomes.

## Conclusion

If the CQI interventions as designed in this study are proven to be effective in improving long-term outcomes of lifelong ART in MCH clinics, they will provide the DRC government and PEPFAR with a scalable strategy to strengthen the quality of the HIV continuum of care and help the country and PEPFAR move towards their aspiration of an AIDS-free generation. Because of the extreme scarcity of resources in DRC settings, successful use of CQI at scale to improve long-term outcomes of therapy in women who start lifelong ART during pregnancy and breastfeeding in this setting will entail that the intervention can be used in virtually all settings.
